# Diagnosis of Guillain-Barré syndrome and use of Brighton criteria in Peruvian hospitals

**DOI:** 10.1590/0004-282X-ANP-2021-0225

**Published:** 2022-08-08

**Authors:** Marco Malaga, Aaron Rodriguez-Calienes, Victor Velasquez-Rimachi, Carlos Alva-Diaz

**Affiliations:** 1 Universidad de San Martín de Porres, Facultad de Medicina Humana, Lima, Peru.; 2 Red de Eficacia Clínica y Sanitaria (REDECS), Lima, Peru.; 3 Universidad Científica del Sur, Grupo de Investigación Neurociencia, Efectividad Clínica y Salud Pública, Lima, Peru.; 4 Universidad Señor de Sipán, Chiclayo, Peru.

**Keywords:** Guillain-Barré Syndrome, Evidence-Based Practice, Evidence-Based Medicine, Síndrome de Guillain-Barré, Práctica Clínica Basada en la Evidencia, Medicina Basada en la Evidencia

## Abstract

**Background:**

Guillain-Barré syndrome (GBS) is an autoimmune disease of the peripheral nervous system that caused multiple epidemiological outbreaks in Peru during 2018 and 2019. It is usually diagnosed using the Brighton criteria (BC).

**Objective:**

We aimed to determine the performance of Peruvian neurologists in diagnosing GBS based on the BC, along with its associated factors.

**Methods:**

This was a retrospective multicenter cohort study. We included patients diagnosed with GBS between 2007 and 2018 in three public hospitals in Lima, Peru. We collected data regarding demographic, clinical and management characteristics. We evaluated the use of the BC for confirmatory diagnosis of GBS and developed a logistic regression model to identify factors associated with its use.

**Results:**

Out of 328 cases, we reviewed 201 available charts. The median age was 48 years, with male predominance. Over half of the patients presented an inadequate motor examination according to their Medical Research Council (MRC) score. Additional testing included lumbar puncture and electrophysiological testing, in over 70% of the cases. The BC showed certainty level 1 in 13.4% and levels 2 and 3 in 18.3%. Neither the quality of the motor examination nor the type of institution showed any association with the BC.

**Conclusions:**

Level 1 diagnostic certainty of the BC was met in less than one quarter of the cases with a GBS diagnosis in three centers in Lima, Peru, between 2007 and 2018. This level was not significantly associated with being treated in a specialized institute, rather than in a general hospital.

## Resumen

**Antecedentes:**

El Síndrome de Guillain-Barré (SGB) es una enfermedad autoinmune del sistema nervioso periférico, causante de brotes epidemiológicos en Perú entre el 2018 y el 2019. El diagnóstico se realiza a través de los Criterios de Brighton (CB).

**Objetivo:**

Determinar el desempeño de neurólogos peruanos en diagnosticar SGB basándose en los CB, así como factores asociados.

**Métodos:**

Cohorte retrospectiva multicéntrica. Incluimos pacientes diagnosticados con SGB del 2007-2018 en 3 hospitales públicos en Lima, Perú. Recolectamos sus características demográficas, clínicas y de manejo. Evaluamos el uso de los CB para el diagnostico de SGB y empleamos un modelo de regresión logística para identificar los factores asociados con su uso.

**Resultados:**

De 328 casos, revisamos 201 historias disponibles. La edad mediana fue 48 años, con predominancia masculina. Mas del 50% de pacientes presento un examen motor inadecuado acorde con el puntaje MRC. Se realizaron exámenes auxiliares como punción lumbar y estudios electrofisiológicos en mas del 70% de pacientes. Se obtuvo un nivel de certeza 1 para los CB en un 13.4% de casos , y un nivel 2 o 3 en un 18.3%. El nivel no estuvo asociado con la calidad del examen motor ni el tipo de institución de atención.

**Conclusiones:**

Un diagnostico nivel 1 de certeza acorde con los BC se obtuvó en menos de un cuarto de casos diagnosticados como SGB. Este nivel no estuvo asociado con la atención en una institución especializada, comparado con un hospital general.

## INTRODUCTION

Guillain-Barré syndrome (GBS) is an autoimmune disease of the peripheral nervous system that presents with axonal or demyelinating neuropathy, with ascendent centrifugal progression. GBS affects around 1.1 patients per 100,000 inhabitants annually around the globe^1^. In Peru, multiple epidemiological outbreaks were reported during 2018 and 2019, which raised the incidence from 0.62 to 0.92 patients per 100,000 inhabitants and led to declaration of a healthcare emergency in five regions of the country^2^.

The first diagnostic criteria for GBS were developed in 1976 and were expanded by Asbury and Cornblath in 1990 ^3^ ^,^ ^4^. However, the criteria elaborated by the Brighton Collaboration have been recommended in national and international clinical-practice evidence-based guidelines since 2010^5^ ^,^ ^6^. These criteria include flaccid limb weakness, areflexia in the affected limbs, a monophasic course of less than 28 days, albuminocytological dissociation in cerebrospinal fluid (CSF), suggestive findings in electrophysiological studies (EPS) and the absence of an alternative diagnosis^7^. 

Use of the Brighton criteria (BC) extends around the world. Countries such as the Netherlands, India, Bangladesh and China have reported that the proportion of patients at diagnostic certainty level 1, which indicates fulfillment of all the BC criteria, was near to or greater than 60%^7^ ^-^ ^10^. A complete diagnostic workup for patients with these criteria is important because they present severe weakness and possible imminent death^9^. However, additional testing such as EPS and CSF studies may be difficult in low-resource settings^9^, which means that it is more likely that the BC would be applied in centers in which these specialized tests are available. 

Here, we aimed to determine the performance of Peruvian neurologists in diagnosing Guillain-Barré syndrome (GBS) based on the Brighton criteria (BC), along with factors associated with GBS, in three Peruvian referral institutions between 2007 and 2018. 

## METHODS

### Patients

We included a retrospective multicenter cohort from three referral institutions in Lima, Peru: the *Instituto Nacional de Ciencias Neurológicas* (INCN), an institute that specializes in neurological diseases; and two national hospitals, the *Hospital Nacional Dos de Mayo* (HNDM) and *Hospital Nacional Arzobispo Loayza* (HNAL). Clinical records from patients diagnosed with GBS between January 1, 2007, and December 31, 2018, were reviewed. We excluded patients for whom clinical records were not available and also those with neuropathy secondary to diabetes mellitus, alcohol intoxication, malignancy or human immunodeficiency virus. 

### Variables

The following variables were analyzed: age, sex, institution, clinical presentation, motor assessment at admission using the Medical Research Council (MRC) score, level of diagnostic certainty according to the BC, length of time between disease onset (from onset of motor symptoms) and obtaining EPS and lumbar puncture (LP) results. 

The level of diagnostic certainty was classified into four levels according to the BC: level 1 fulfills all diagnostic criteria; level 2 fulfills all clinical parameters, without the final results from LP and EPS; level 3 fulfills only clinical parameters; and level 4 does not fulfill the criteria of level 3, but all other diagnoses are excluded (Table 1)^7^. 

**Table 1.** Diagnostic criteria and level of diagnostic certainty for Guillain-Barré syndrome.

Diagnostic criteria	Level of diagnostic certainty			
	1	2	3	4
Bilateral and flaccid weakness of limbs	+	+	+	+/-
Decreased or absent deep tendon reflexes in weak limbs	+	+	+	+/-
Monophasic course and time between onset and nadir of 12 h to 28 days	+	+	+	+/-
CSF cell count < 50/ml	+	+^a^	-	+/-
CSF protein concentration > 0.45 g/L	+	+/- ^a^	-	+/-
NCS findings consistent with one of the subtypes of GBS	+	+/-	-	+/-
Absence of alternative diagnosis for weakness	+	+	+	+

a If CSF is not collected or results not available, nerve electrophysiology results need to be consistent with the diagnosis of Guillain-Barré syndrome; +: present; -: absent; +/-: present or absent; CSF: cerebrospinal fluid; NCS: nerve conduction studies; GBS: Guillain-Barré syndrome.

The MRC score establishes a score of 0-5 for each muscle group, with an overall maximum score of 60 ^11^. The quality of the motor examination is categorized as “complete” if at least 6 of the 12 muscle groups included in the MRC score were assessed (necessarily more than three muscle groups for each hemibody). It is considered “incomplete” in the remaining cases ^11^. 

The time between disease onset and LP was categorized as ≤ 7 days or > 7 days, whereas for EPS the cutoff point was 14 days, in accordance with the Peruvian guidelines for diagnosis and treatment of patients with GBS^6^. We categorized the facilities at which care took place into two groups: national hospital (HNAL or HNDM) and specialized institute (INCN), taking into account the differences in the capacity and expertise for management of neurological diseases. 

### Statistical analysis

STATA version 16.0 was used for the analysis. For quantitative and qualitative variables, measurements of statistical dispersion and frequency were used, respectively. Categorical data for each institution were compared using the chi-square test if normally distributed and the Fisher exact test if not normally distributed. A logistic regression model was used to determine whether clinical characteristics (cranial nerve involvement, dysautonomia and electromyographic subtype) or care-related characteristics (care facility, quality of motor examination and length of time until LP or EPS) were associated with use of the BC to confirm the diagnosis with certainty level 1. These factors were entered into the model in a stepwise fashion if they had a p-value less than or equal to 0.2. 

This study was approved by the Institutional Review Boards of the three participating institutions (INCN-IRB, HNDM-IRB and HNAL-IRB) before data collection. The confidentiality of participants' identities was maintained. 

## RESULTS

We identified 328 GBS cases and included 201 patients whose charts were available for review. The median age was 48 years (interquartile range [IQR]: 18-86), and 54.2% were male. Among the 201 patients, 86.2% presented bilateral flaccid weakness at admission, 90% had a monophasic course of disease (< 28 days) and 45.2% had areflexia in the affected limbs. Cranial nerve involvement and dysautonomia were present in 39.2% and 13.4% of patients, respectively. The axonal and demyelinating subtypes were also observed in 64.6% and 35.4% of the patients, respectively (Table 2).

**Table 2.** Clinical characteristics of patients diagnosed with Guillain-Barré syndrome.

Clinical characteristics of patients		Fr	%	N (%)	
				Hospital ^†^	Institute
Sex	Female	92	45.8	57 (40.4)	35 (58.3)
	Male	109	54.2	84 (59.6)	25 (41.8)
Monophasic course < 28 days	No	20	10.1	11 (7.9)	9 (15)
	Yes	179	90	128 (92.1)	51 (85)
Bilateral and flaccid weakness	No	26	13.8	18 (14)	8 (13.6)
	Yes	162	86.2	111 (86.1)	51 (86.4)
Areflexia in weak limbs	No	103	54.8	72 (55.8)	31 (52.5)
	Yes	85	45.2	57 (44.2)	28 (47.5)
Cranial nerves affection	No	121	60.8	83 (59.7)	38 (63.3)
	Yes	78	39.2	56 (40.3)	22 (36.7)
Dysautonomia	No	174	86.6	116 (82.3)	58 (96.7)
	Yes	27	13.4	25 (17.7)	2 (3,.3)
Increased protein in CSF	No	46	30.5	29 (27.9)	17 (36.2)
	Yes	105	69.5	75 (72.1)	30 (68.8)
Normal CSF cell count	No	1	0.6	1 (0.9)	0 (0)
	Yes	155	99.4	106 (99.1)	49 (100)
Albuminocytological dissociation	No	47	31.1	30 (28.9)	17 (36.2)
	Yes	104	68.8	74 (71.2)	30 (63.8)
Electrophysiological subtype	Demyelinating	51	35.4	29 (31.6)	22 (42.3)
	Axonal	93	64.6	63 (68.5)	30 (57.7)
Total				141 (70.2)	(29.9)

† Hospital Nacional Dos de Mayo and Hospital Nacional Arzobispo Loayza; CSF: cerebrospinal fluid; Fr: frequency.

According to the BC, the proportion of confirmed cases (certainty level 1) was 13.4% and the proportion of suspicious cases (certainty levels 2 and 3) was 18.3%. The remaining 68.3% of the patients met level 4 of certainty. There was no statistically significant difference between the institutions at any of the certainty levels (p = 0.396). Most patients at certainty level 4 met most of the clinical criteria except for altered tendon reflexes (84.3%) (Table 3). 

**Table 3.** Brighton criteria and diagnostic certainty level among patients diagnosed with Guillain-Barré syndrome.

	BC	Certainty level			
		1	2	3	4
Bilateral and flaccid weakness of limbs	No	0 (0.00%)	0 (0.00%)	0 (0.00%)	26 (21.67%)
	Yes	29 (100.00%)	30 (100.00%)	7 (100.00%)	94 (78.33%)
Decreased or absent deep tendon reflexes	No	0 (0.00%)	0 (0.00%)	0 (0.00%)	102 (84.30%)
	Yes	29 (100.00%)	30 (100.00%)	7 (100.00%)	19 (15.70%)
Monophasic course with 12 h to 28 days from onset to nadir	No	0 (0.00%)	0 (0.00%)	0 (0.00%)	20 (16.53%)
	Yes	29 (100.00%)	30 (100.00%)	7 (100.00%)	101 (83.47%)
Normal CSF cell count	No	-	-	-	-
	Yes	29 (100.00%)	17 (100.00%)	-	99 (100.00%)
Increased CSF protein concentration	No	0 (0.00%)	7 (43.75%)	-	37 (38.54%)
	Yes	29 (100.00%)	9 (56.25%)	-	59 (61.46%)
NCS findings consistent with one subtype	No	0 (0.00%)	10 (33.33%)	7 (100.00%)	35 (28.93%)
	Yes	29 (100.00%)	20 (66.67%)	0 (0.00%)	86 (71.07%)

BC: Brighton criteria.

In the three institutions, a mean proportion of 35.8% of the patients was adequately examined using the MRC score. At the specialized institute, this percentage was 78.3%, with a statistically significant difference compared with the national hospitals (p < 0.000) (Table 4).

**Table 4.** Characteristics of a diagnosis of Guillain-Barré syndrome.

Characteristics		Fr	%	N (%)		p-value
				Hospital	Institute	
Level of diagnostic certainty	1	25	13.4	17 (13.3)	12 (20.3)	0.396^‡^
	2	28	15.1	23 (18)	7 (11.9)	
	3	6	3.2	6 (4.7)	1 (1.7)	
	4	127	68.3	82 (64.1)	39 (66.1)	
Motor examination	Incomplete	129	64.2	116 (82.3)	13 (21.7)	< 0.000*^,†^
	Complete	72	35.8	25 (17.7)	47 (78.3)	
Lumbar puncture	No	52	25.9	40 (28.4)	12 (20)	0.215 ^†^
	Yes	149	74.1	101 (71.6)	48 (80)	
Time until lumbar puncture	Early (≤ 7)	111	76	74 (74.8)	37 (78.7)	0.559 †
	Late (> 7)	35	24	25 (25.3)	10 (21.3)	
Electrophysiological studies	No	47	23.4	42 (29.8)	5 (8.3)	0.001*^,†^
	Yes	154	76.6	99 (70.2)	55 (91.7)	
Time until electrophysiological studies	≤ 14	96	62.8	62 (63.3)	34 (61.8)	0.859 ^†^
	>1 4	57	37.3	36 (36.7)	21 (38.2)	

*p < 0.05; ^†^ Chi-square test; ^‡^ Fisher exact test; FR: frequency.

An LP was performed on 74.1% of the patients, among which 76% of the procedures were carried out within the first seven days after admission. No significant differences were observed between the care facilities (p = 0.559). EPS was performed on 76.6% of patients and was used more frequently in the specialized institute (91.7%; p = 0.001). In 62.8% of the cases, EPS was carried out within 14 days after admission. 

In the bivariate analysis, patient age and the timing of LP and EPS showed p-values greater than the cutoff. In multivariate logistic regression, we found that use of both early LP (< 7 days) and late EPS (> 14 days) increased the likelihood of application of the BC for confirmatory diagnosis. The remaining clinical or care characteristics were not significant (Table 5). 

**Table 5.** Factors associated with application of the Brighton criteria with diagnostic certainty level 1 for Guillain-Barré syndrome.

Variables	Brighton criteria diagnostic certainty level 1					
	No	Yes	Crude PR (95% CI)		p	Adjusted PR (95% CI)
	N (%)	N (%)				
Institution	National hospital	111 (86.7)	17 (13.3)	Ref	0.218	Ref
	Specialized institute	47 (79.7)	12 (20.3)	1.67 (0.74 - 3.76)		0.73 (0.23 - 2.35)
Age^†^	< 65	132 (83.0)	27 (17.0)	Ref	0.200	Ref
	≥ 65	26 (92.9)	2 (7.1)	0.38 (0.08 - 1.68)		0.28 (0.03 - 2.39)
Sex	Female	69 (82.1)	15 (17.9)	Ref	0.424	Ref
	Male	89 (86.4)	14 (13.6)	0.72 (0.33 - 1.60)		^‡^
Complete medical research council	No	101 (87.1)	15 (12.9)	Ref	0.216	Ref
	Yes	57 (80.3)	14 (19.7)	1.65 (0.75 - 3.67)		1.29 (0.42 - 4.01)
Cranial nerve involvement	No	97 (86.6)	15 (13.4)	Ref	0.311	Ref
	Yes	60 (81.1)	14 (18.9)	1.51 (0.68 - 3.35)		^‡^
Dysautonomia	No	136 (83.4)	27 (16.6)	Ref	0.309	Ref
	Yes	22 (91.7)	2 (8.3)	0.46 (0.10 - 2.06)		^‡^
Lumbar puncture	Early	80 (76.9)	24 (23.1)	Ref	0.090	Ref
	Late	30 (90.9)	3 (9.1)	0.33 (0.09 - 1.19)		0.16 (0.04 - 0.65)
Electrophysiological studies	Early	78 (83.9)	15 (16.1)	Ref	0.197	Ref
	Late	39 (75.0)	13 (25.0)	1.73 (0.75 - 4.00)		3.46 (1.20 - 9.97)
Electrophysiological subtype	Demyelinating	36 (76.6)	11 (23.4)	Ref	0.691	Ref
	Axonal	70 (79.5)	18 (20.5)	0.84 (0.36 - 1.97)		^‡^

†Median ± SD; ‡Variables did not require adjustment; MRC: medical research council; LP: lumbar puncture; EPS: electrophysiological studies.

## DISCUSSION

This study assessed the diagnostic management of GBS and use of the BC in three Peruvian institutions between 2007 and 2018. We found that level 1 diagnostic certainty was met in only 13.4% of the GBS cases, and complementary tests were used in the cases of 75% of the patients. Likewise, more than half of the patients presented an incomplete motor examination using the MRC score. 

The proportion of patients with affected reflexes in our cohort was lower (45%) than what was reported in a previous Peruvian study (84%)^12^. It was also the main clinical criteria missing among patients with certainty level 4. This difference may have been a consequence of inadequate examination, inadequate recording or “normal” reflexes, which have been associated with higher frequency of the axonal variant of GBS, as in our cohort. Although there is still divergence of opinions regarding the predominant variant in Latin America, there are reports from pediatric cohorts showing that the axonal subtype made up to 40-65% of the cases of GBS. This stands in contrast to findings from Europe and North America, where AIDP has a frequency of 60-80%^13^ ^,^ ^14^. However, we did not observe any association between the electrophysiological variant and use of the BC with level 1 diagnostic certainty. 

In the present study, the rate of application of the BC for GBS diagnosis with level 1 diagnostic certainty was lower (13.4%) than in studies conducted in the Netherlands, India and Bangladesh, which met the criteria for level 1 in 61%, 62% and 58% of the patients, respectively^7^ ^-^ ^9^. This finding might be explained by lack of knowledge of these criteria and the recommendations for its use, or by physicians’ disagreement with their use^15^ ^,^ ^16^. In addition, the lower proportion of Peruvian neurologists, in contrast with the World Health Organization recommendations, may have contributed to lower use of the BC^17^. Complementary tests such as LP and EPS were frequently used (in around 75% of the cases) in our study: thus, availability does not seem to have been an influencing factor. 

The quality of motor examination with the MRC score was incomplete in most patients (64.2%), while complete quality of examination predominated in the specialized institute (78.3%). A higher proportion of neurologists with greater experience of using these scores could likely explain this finding^18^ ^,^ ^19^. 

CSF analysis is helpful for confirming the diagnosis and for ruling out another differential diagnosis^20^. Most of our patients (76%) underwent LP during hospitalization, within seven days of disease onset. An early LP shows albuminocytological dissociation in 50-66% of GBS patients, and this proportion rises to 75% of the cases if the procedure is performed more than three weeks after disease onset. Thus, it is recommended that this test is repeated if negative^21^. Since most LPs in our study were performed within the first seven days, during which the hallmark findings of GBS are typically less frequently found, this could explain the low fulfillment of the BC among these patients.

EPS findings reinforce the diagnosis and allow differentiation of the variants of GBS^22^. The relevance of performing EPS after the second week of the disease lies in the fact that more than 85% of patients present consistent signs of GBS after this time^23^. We observed that after 14 days, EPS was less frequently used (37.3%). This could be a consequence of patients’ refusal to undergo the procedure^24^, lack of consideration of this test among neurologists or lack of availability of this equipment in the public sector^25^. 

We found that being treated in a specialized institution was not associated with a higher rate of certainty level 1 of GBS diagnosis, despite the greater use of LP, EPS and complete motor examinations. Apart from these institutional factors, none of the patient-related factors assessed showed any association. We believe that physicians’ familiarity with and acceptance of the BC should be explored in order to determine whether these are associated with the lower rate of use of the BC observed in our population.

Our study was limited by lack of access to patient records, due to unavailability of old paper records in one of the centers. However, our sample still had sufficient power and, as the only common factor among the factors excluded was the date on which these patients were treated, we do not believe that this resulted in a high risk of selection bias. Likewise, due to the retrospective design of this study, there was a risk of bias in data collection, which we reduced by using strict case definitions, standardized case report forms and exclusion of cases with missing data from the univariate analysis.

In conclusion, level 1 diagnostic certainty of the BC was met in less than one quarter of the cases with a GBS diagnosis between 2007-2018 in three national centers in Lima, Peru. This level was not significantly associated with being treated in a specialized institute, compared with a general hospital. Additionally, less than half of the patients presented a complete motor evaluation using the MRC score. Further research should assess whether neurologists’ preferences or institutional factors can explain the low use of the BC and how this can be increased. 
